# Ultimate Bearing Capacity Analysis of CFRP-Strengthened Shield Segments Using Bonding Slip Behavior Experiments

**DOI:** 10.3390/ma13184200

**Published:** 2020-09-21

**Authors:** Hong-bin Nie, Shuan-cheng Gu

**Affiliations:** 1 School of Architecture and Civil Engineering, Xi’an University of Science and Technology, Xi’an 710054, China; gsc5583297@xust.edu.cn; 2 Department of Rail Engineering, Shaanxi Railway Institute, Wei Nan 714000, China

**Keywords:** carbon-fiber-reinforced plastics (CFRPs), hygrothermal effect, slip shield segments, ultimate strength

## Abstract

Shield segments of subway tunnels are often exposed to the combined actions of several hygrothermal factors that could lead to accidents such as water seepage and tunnel collapse. Further, they often break and deform owing to formation pressure. In addition, uncertainties related to the stress relaxation characteristics and bonding performance of carbon-fiber-reinforced plastics (CFRPs) under a hygrothermal environment make their application in subway systems difficult. This study analyzes the effects of the slip-on-bending strength of CFRP-strengthened shield segments in a hygrothermal environment. In the study, the shield segments are damaged at ambient pressure under a combination of humidity (0%, 5%, and 10%) and temperature (20 °C, 25 °C, 30 °C, and 40 °C). An experimental procedure is designed to evaluate a CFRP-reinforced concrete arch. The method predicts the load–slip relationship and maximum shearing stress and strain. Moreover, confined compression tests are conducted on a tunnel segment lining strengthened with CFRP to evaluate the bearing capacity of the CFRP-strengthened shield segments. An equation for the latter’s ultimate bearing capacity is developed based on the elastic layer system theory, stress boundary condition, and bending stress characteristics of axisymmetric elements. It was found that the results from the developed model are compared with the experimental values of CFRP-strengthened shield segments under different humidity values (0%, 5%, and 10%) and a constant temperature. The ultimate strength—the debonding deflection of the CFRP-strengthened shield segment—can be predicted using the proposed ultimate bearing capacity equation with sufficient accuracy.

## 1. Introduction

The accelerated increase in urban economic integration has resulted in the rapid development of construction technology related to urban rail transit (subways). Several shield construction methods that can be applied to subway engineering have been developed [1]. Segmental tunnel linings often break and deform, which can lead to accidents such as water seepage and collapse [2]. A technique for strengthening the shield comprises repouring concrete after embedding the steel bars [3,4,5]. This technique requires substantial work and is prone to hazard events during the strengthening process. Another technique consists of bonding aramid fiber sheets that age easily. However, structures thus obtained have insufficient strength, and the process of grouting in the back produces a hole [6,7,8,9]. Therefore, there is an urgent need to replace the materials used in strengthening techniques; specifically, materials having good durability and high strength are required.

Carbon-fiber-reinforced plastics (CFRPs) are multifunctional composite materials with properties such as high strength, antivibration, high toughness, and good durability [10,11,12,13]. Hence, CFRPs have been widely used in engineering practice (e.g., in bridges and building structures). However, as segmental linings for subways operate under humid and high-stress environments, employing the technology used for CFRP strengthening during subway construction is challenging [14,15,16]. The mechanical performance of CFRP-reinforced segmental linings depends on the reliability of the bonding between the CFRP and the surrounding concrete in a hygroscopic environment [17], which is referred to as hygrothermal aging [18].

Previous reports considering the durability of CFRP in complex environments such as crude oil and seawater found that the ultimate bearing capacity of an RC (Reinforced Concrete) column bonded with CFRP was unaffected, the displacement decreased by 37% and 53%, and the ductility gradually decreased between 1000 h and 1500 h, respectively [19,20]. Moreover, direct pull-out tests, which indicate that CFRP bars are embedded in concrete, showed a decrease of 12% in the ultimate bearing capacity caused by wet–dry cycles, with similar characteristic results for the ‘‘fish-spine’’ crack pattern. In addition, the bonding behavior [21,22] of concrete with CFRP under load and seawater wet–dry cycles where the entire liquid was 5% NaCl dropped from 85% to 60%. In contrast, in [23], the joints of the CFRP/steel under wet–freeze cycles showed no negative effect. Interestingly, the corresponding strength was lower in hot/wet environments [24]. The strain distribution on the CFRP-strengthened beams showed that overloading fatigue and wetting–drying cycles caused considerable fatigue damage, as represented by the sign D [25]. A single-lap direct-shear test showed that the bond strength of CFRP first increased and then decreased with an increment in aging until it finally reached a constant value. In other words, the strength of the epoxy resin is not significantly affected between 1000 and 1500 h. In [26], the dynamic bond-slip behavior of CFRP–concrete was studied using a single-lap shear test focusing on dynamic debonding behavior. The results showed that the dynamic bond-slip behavior of CFRP–concrete is sensitive to the dynamic response of concrete. The rates were between 3900 mm/s and 1927 mm/s [26,27]. The CFRP bonding performance was affected in different environments, especially in hygroscopic conditions.

**Figure 1:**
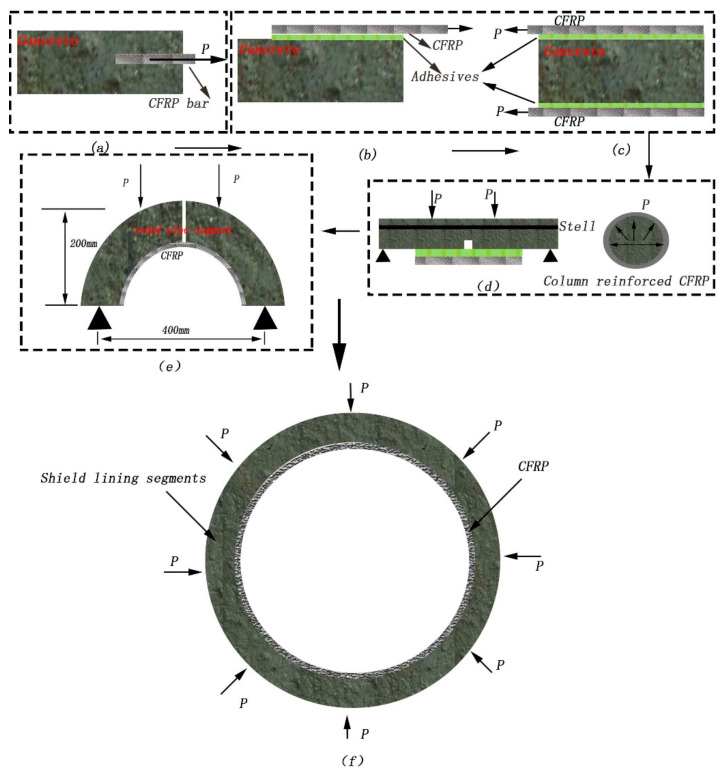
Design of problem statement. (**a**) Embedded methods, (**b**) single-shear method, (**c**) double-shear method, (**d**) reinforced notched beam, (**e**) bending and shear test, (**f**) stress model of this study.

The related literature indicates that experiments such as the single-sided partial-shear test or double-sided shear test are used to evaluate the bonding performance of CFRP. Both experiments are destructive tests and they employ CFRP cast inside the concrete that is stretched by a tensile testing machine, as shown in Figure 1a [20]. For a single-sided partial-shear test, two CFRP sheets or CFRP–concrete bonds are directly stretched. In this arrangement, the CFRP is subjected to eccentric tension, as shown in Figure 1b [23,28]. The double-sided shear test refers to CFRP sheets bonded in the upper and lower surfaces of concrete, which is complicated to use in the existing tensile test machine, as shown in Figure 1c [21,24]. Hamada and Fang [29] use the method of the bending shear experiment that is based on the bending experiment of the beam. This test is simple and convenient.

Although various scholars used a series of methods to study the mechanical properties of different CFRP-reinforced structures in complex environments, these studies cannot satisfy the requirements for subway constructions. To apply CFRP in the subway structure, we need to study the bond-slip behavior for CFRP-strengthened segmental lining whose surfaces are arcs surrounded by humidity in a better manner. Further, several scholars have not considered the effect of the reinforcement ratio, which has a considerable effect on concrete structure ductility, when considering CFRP bonding properties.

This study investigates the effects of hygroscopic factors on the mechanical behavior of a concrete arc bonded with CFRP considering the effect of steel. The bond-slip behaviors of the CFRP and concrete are studied through bending-shear experiments, which are improved considering the bending test proposed by Hamada and Fang by transforming the structure surface in Figure 1d. Further, we propose an equation for calculating the bearing capacity of CFRP-reinforced shield according to the relationship between slip and shear force established in the experiments. Finally, the correctness of the theoretical equation is verified via experiments and practical applications of the CFRP-strengthened segmental linings. Finally, a new method for subway repairing is presented.

## 2. Experimental Procedure

### 2.1. Experimental Design

We considered the effect of steel on the behavior of CFRP bonding, inspired by the model of CFRP-reinforced beams and columns. Two arc-shaped sections—created according to the shrinking model of the shield segment—were set up to study the mechanical behavior of the concrete bonded by CFRP under the coupling action of tension and bending moment shown in Figure 1e. Following the research on the bond performance of CFRP-reinforced concrete, the concrete arc CFRP-reinforced shield segment was designed to study the rate of increase in the mechanical performance index, as shown in Figure 1f.

### 2.2. Mechanical Properties of Materials

In this study, the materials included the adhesive used in [30], CFRP, and concrete. The mechanical properties of CFRP (JGN) were measured experimentally based on the China National Standard (GB50367-2013). A special adhesive for structured bonding (JGN-C-china) was used; the mechanical parameters of the CFRP and adhesive are listed in Table 1. The complete results were used as a reference to study the effect of bonding on CFRP.

A mixed proportion of curved specimens was designed according to the specifications of a subway in South China. The concrete mix design was 300: 728: 1093: 185 (cement: sand: gravel: water) in 1 m^3^, produced for C30 concrete as suggested by the China National Standard (GB 50010-2010). Portland cement (P.S.42.5) was used, and its loss-on-ignition and specific surface values were 4.46% and 325, respectively; these values have a strong effect on adhesive bonding. The aggregate was fine river sand, with a complete fineness modulus of 1.97. The gradation of the gravel was in the range of 5 mm–12 mm, where its maximum diameter did not exceed 20 mm.

### 2.3. Experimental System and Method

The experimental system comprised two parts: the slip test of the CFRP-reinforced arch concrete sections and the confined compression strength test of the CFRP-strengthened tunnel segmental lining.

#### 2.3.1. Slip Test of CFRP-Reinforced Concrete Arch Sections

Sixty CFRP-reinforced concrete arch sections were manufactured, as shown in Figure 1e. The concrete arch sections consisted of C30 plain concrete, 500 mm long, 200 mm wide, and 100 mm high. They had a 200 mm crown with an arc length of S = 1257 mm. According to the China National Standard (GB50367-2013) and the actual test scenario, the bonding CFRP was applied by adhesive. Subsequently, the CFRP was repeatedly rolled on the top of this adhesive. The experiment was considered completed when the adhesive appeared on the CFRP surface.

**Figure 2:**
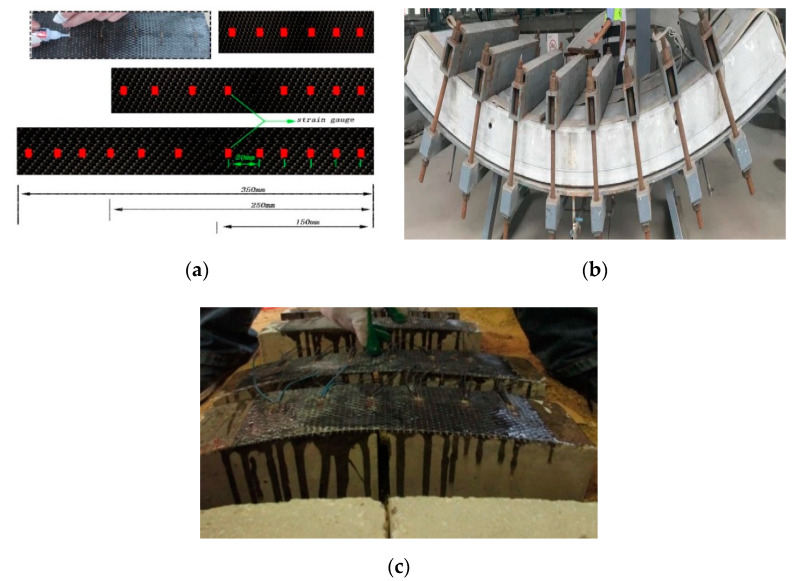
Location of strain gauge paste and rectangular CFRP. (**a**) Resistance strain gauge, (**b**) circular concrete pipe, (**c**) sticking CFRP.

To accurately measure the stress and strain of the CFRP and the concrete under the coupling action of differential hygrothermal environments, we set up electric resistance strain and stress gauges in deferential locations of the CFRP and the concrete surface, as shown in Figure 2. Based on the theoretical knowledge regarding the subway project in southern Chinese cities with temperatures between 12 and 40 °C and a humidity of 3.5–10%, the length and layers of CFRP and the thermal parameters were set at 3.5%, 5%, 10% humidity, as summarized in Table 2.

A press with a back connection provided the bending pressure necessary for the slip test, with a stroke monotonically increasing at a rate of 0.05 mm/min. When it reached 80% of the ultimate load, the loading mode was changed from displacement to load, which increased the rate to 0.05 kN/min.

#### 2.3.2. Confined Compression Strength Test of CFRP-Strengthened Tunnel Segmental Lining

**Figure 3:**
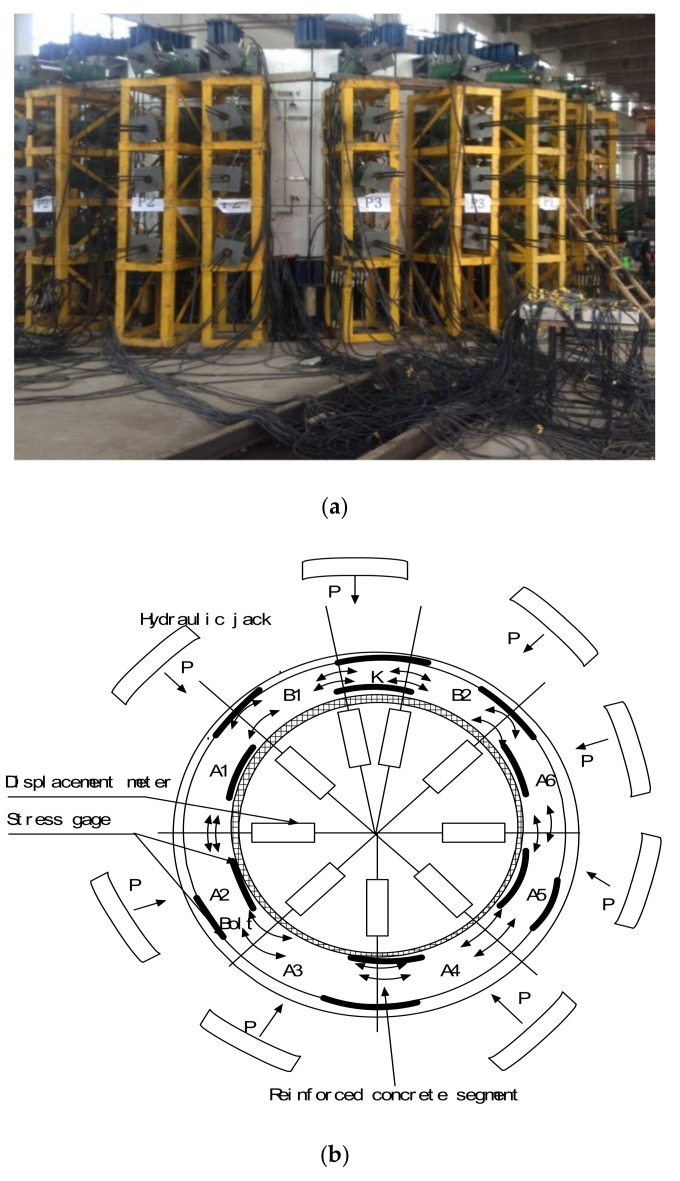
Accelerated loading of CFRP-strengthened tunnel segmental lining. (**a**) Test loading diagram, (**b**) schematic diagram.

The CFRP and concrete bond properties were studied using a Part 1 test: the slip test of the CFRP-reinforced concrete arch sections. According to the fundamental relationship between the shear stress and the slip, a confined compression strength test of the CFRP-strengthened tunnel segmental lining was performed to study the improvements in the bearing capacity of the CFRP-reinforced shield tunnel. The lining of the shield tunnel in this test comprised six wedge-shaped assembly parts, a shape commonly used for shield construction. A tunnel segmental lining with an internal diameter of 6000 mm, an external diameter of 5400 mm, and a gauge of 300 mm was cured in laboratory conditions at 30 °C ± 2 °C, as shown in Figure 3.

## 3. Results and Discussion

### 3.1. Slip Test Description

**Figure 4:**
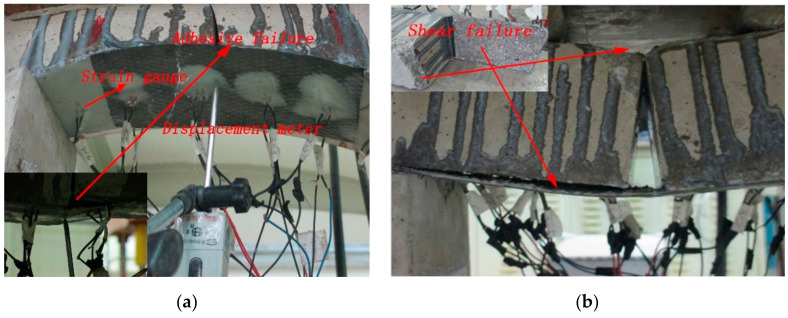
Typical failure mode of CFRP arch concrete. (**a**) The loading process, (**b**) the loading failure.

The typical flexural failure of the CFRP-strengthened concrete arch specimens showed the debonding behavior of CFRP under different circumstances, as shown in Figure 4. For all strengthened specimens, the debonding behavior of CFRP and concrete developed in their adhesive bond as the load was increased. When the load was applied, the strengthened specimens produced a sound corresponding to the cracking of the adhesive and CFRP. Moreover, the load reached approximately 20% of the limit load as the sound increased, and the specimens began to rotate on their short sides in close contact with the axis. With a further increase in the applied load, the angle increased. Finally, the debonding of CFRP and concrete occurred; this damaged model is named the shear-flexural method.

Table 3 lists the typical ultimate loads in the CFRP-strengthened concrete arch structure. The table also provides possible debonding failure locations. With an increase in the CFRP length and layers, a slight increase in the debonding loads was observed; the debonding loads also decreased gradually with an increase in humidity and temperature.

### 3.2. Confined Compression Strength Test Description

**Figure 5:**
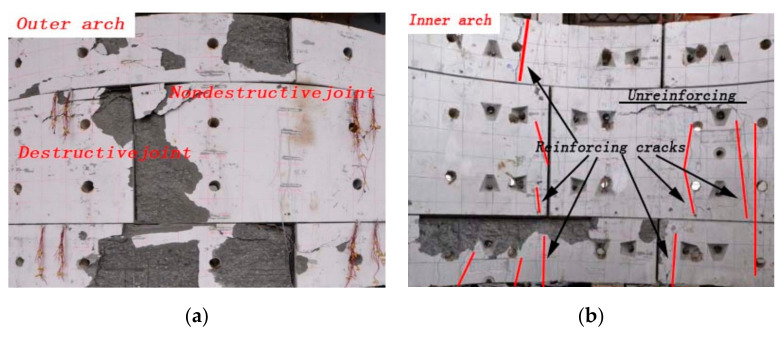
Typical failure mode of a tunnel segmental lining. (**a**) The failure of outer arch, (**b**) the failure of inner arch.

When the segmental tunnel linings were compressed up to 80%, they were bonded with CFRP and cured for seven days. Subsequently, the load continued until the CFRP-strengthened segmental linings produced a sound similar to that in the CFRP slip test. Meanwhile, the outer and inner arches of the segmental linings showed large cracks developed mostly along the main tunnel axis. Opposite to the cracks of the usual tunnel segmental linings, the crack distributions reflected the CFRP effect. Crack distributions in the segmental linings under different humidity and loading levels are shown in Figure 5.

## 4. Parameter Analysis

### 4.1. Load Analysis on Bond-Slip Behavior

**Figure 6:**
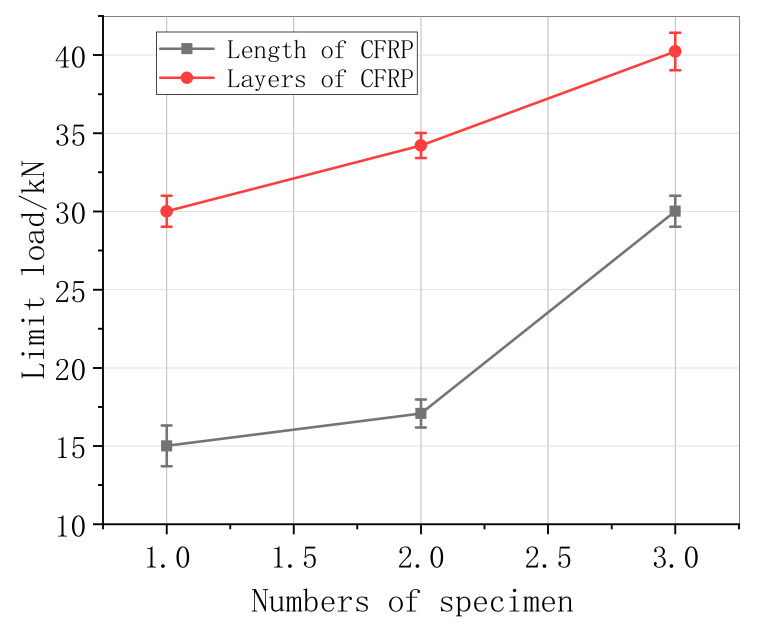
Loading of the CFRP parameters.

Figure 6 and Table 3 show the results for the CFRP-strengthened specimens in the control group that were poured and cured under standard conditions. The specimen limit load for specimen 1-3 was slightly different compared to specimen 1-1. Moreover, the load for specimen 1-3 increased by 101%, caused by an increase in the CFRP cross-bonding area and concrete after the length of the CFRP was increased from 150 mm to 350 mm. The limit load for specimen 1-3-3 increased by 29.9% compared with that for the 1-3 specimen. The results indicate that increasing the length then increases the number of layers in terms of the load.

**Figure 7:**
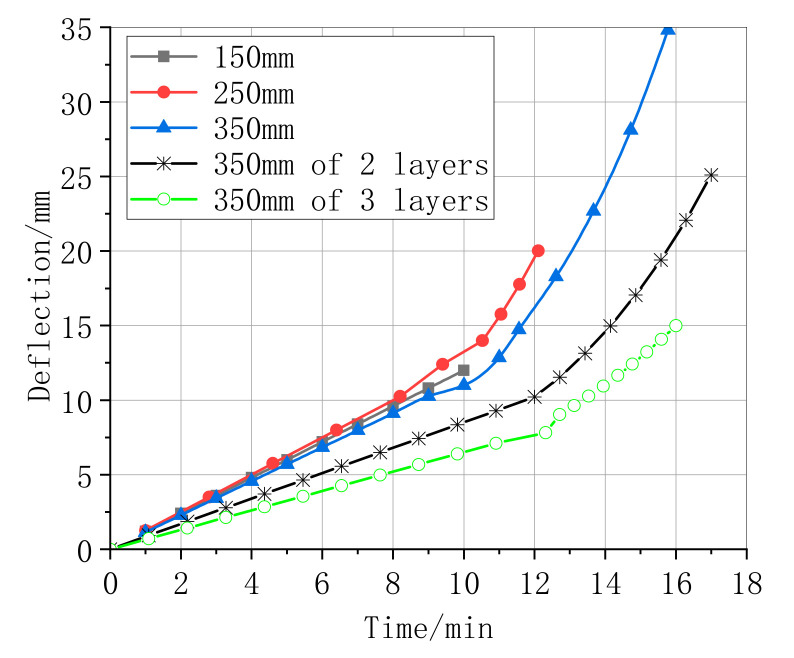
Deflection of the CFRP parameters.

Figure 7 shows the limit load corresponding to a central displacement for all standardly strengthened specimens. With an increase in the CFRP length, the displacement increased accordingly. Moreover, the upper limit of the 350 mm length of the CFRP was 35 mm, which was the maximum. When the specimen was first loaded, the displacement increased linearly, with a further increment in the load. The displacement of the specimens’ bonding lengths 350 mm and 250 mm of the CFRP increased to the limit rapidly. This clearly indicates that the strengthened specimens cause the debonding of the CFRP. While these data of the bonding layers of CFRP increased more gradually with an increase in CFRP length compared with the number of layers, the displacements of the strengthened specimens reduced with an increase in the added number of layers of the CFRP.

**Figure 8:**
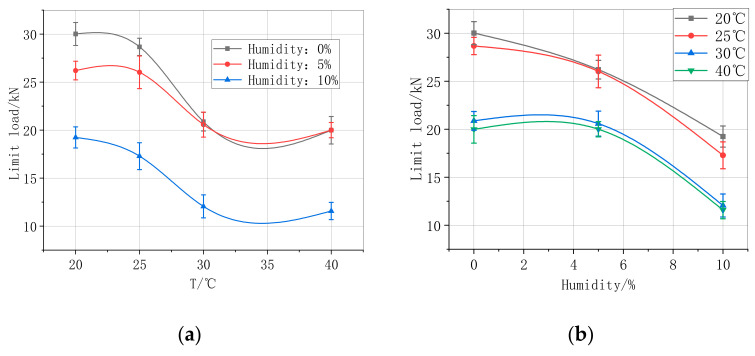
Limit load of CFRP parameters. (**a**) Temperature–load relationship under different humidity, (**b**) humidity–load relationship under different temperature.

Figure 8a shows the failure load of specimens tested at 20 °C, 25 °C, 30 °C, and 40 °C, while Figure 8b presents the failure load of the specimens measured in humidity conditions of 0%, 5%, and 10%. The increase in temperature caused a significant decrease in the carrying capacity of the strengthened specimens, especially when the temperature was near and above 30 °C. However, the carrying capacity of the strengthened specimens increased when the temperature was near 40 °C. This convincingly demonstrates that the limit load decreases very slowly when reducing the temperature to a particular level. In other words, the temperature has a certain effect on the bearing capacity; however, the effect is finite. The effect of humidity between 0% and 10% is not similar to that of the temperature factor. The limit load reduces, which is considerably lower with an increase in humidity.

Based on the relationship between the limit load, temperature, and humidity in Figure 8a,b, the relationship between the limit load and the temperature follows the Boltzmann criterion function. This relationship can be defined by

(1) $$P=\frac{A_{1}-A_{2}}{1+e^{\left(T-T_{0}\right)}}+A_{2}$$

In contrast, the relationship between the load and humidity can be described by the ExpAssoc equation, which is given as

(2) $$P=P_{0}+\sum_{i=1}^{2}A_{i}(1-e^{-H/t_{i}})$$

where P and T are the maximum applying limit load and curing temperature, respectively, T_0_ is the base value of temperature, A_1_ is the coefficient of the first deflecting drop, and A_2_ is the coefficient of the second deflecting drop. Table 4 lists the coefficients of fatigue. P_0_ is the modified loaded parameter, H is expressed by humidity, i is the step counter, A is the humidity enlargement coefficient, and t is the degree of curve descent. The summary of coefficients, including the limit load, steps, and other parameters, is listed in Table 5.

**Figure 9:**
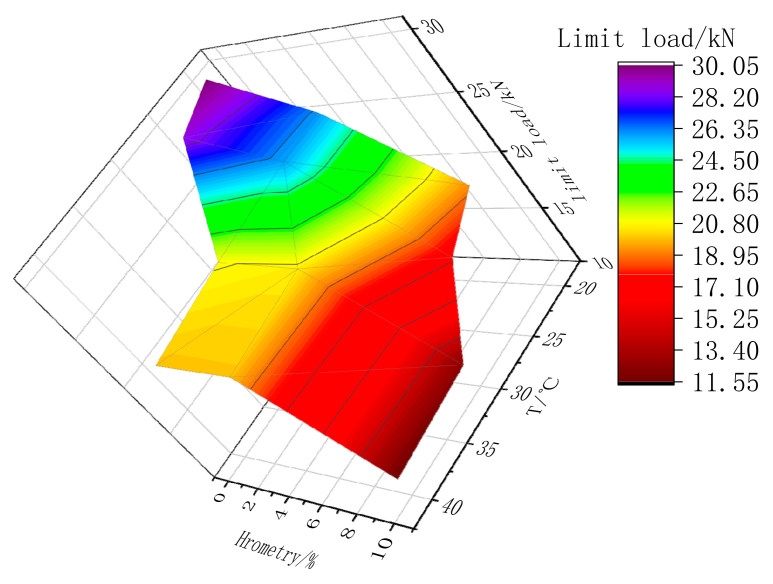
Limit loads under hygrothermal coupling.

The limit load on tested specimens under hygrothermal coupling is shown in Figure 9. In strengthened specimens, the lower limit load was set to 10 kN to show the responses of the limit load clearly. For all specimens, the load decreased substantially after coupling at a temperature of 20 °C and humidity of 0%. With a further increment in humidity (up to 5%), the limit load exhibited a sharp steady as shown in Figure 9 (yellow), which corresponds to the debonding load of 50%. Following the first steady, another quick drop appeared in Figure 9 (red) when the humidity increased from 5% to 10%, thereby revealing the further damage evolution of the strengthened specimens.

### 4.2. The Maximum Shearing Stress Analysis

**Figure 10:**
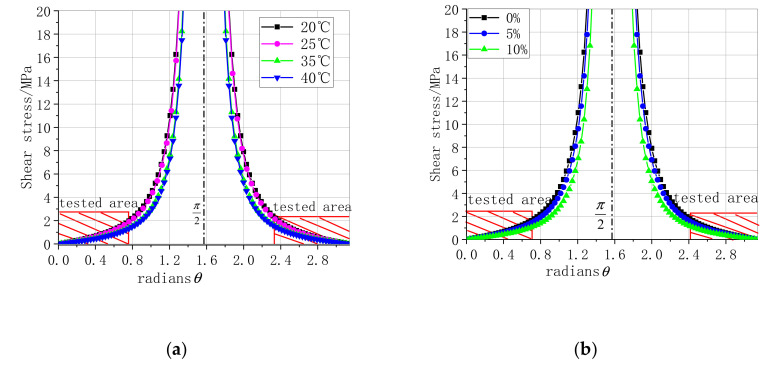
Relationship of shear stress and slope under different values of T and humidity. (**a**) Relationship between T and θ, (**b**) relationship between humidity and θ.

In this study, the high h of the specimen was 100 mm and the central displacement d is shown in Figure 7. This study of the slope does not consider the effective bond length ratio; the ratio is $\frac{l_{e}}{l_{}}=1$ and as the load increased further, the two strengthened specimens rotated by the supporting point, thus, the maximum shear had a great matter with the slope (θ) in Appendix A [30]. The relationship of the maximum shear and slope (θ) under different temperature and humidity values when the load reaches the limit under different scenarios is shown in Figure 10. The test results indicated that specimens fail before the slope is 0.75, which indicates that the slope has little effect on the maximum shear stress. Subsequently, the maximum shear can be expressed by Equations (3) and (4) under different temperature and humidity values according to Equations (1) and (2).

(3) $$\tau_{1\max}=0.5(\frac{A_{1}-A_{2}}{1+e^{\left(T-T_{0}\right)}}+A_{2})\frac{\sin(\theta )}{\left(1-2\sin^{2}(\theta )\right)}\times \frac{l}{l_{e}w(h+d)}$$

(4) $$\tau_{1\max}=0.5(P_{0}+\sum_{i=1}^{2}A_{i}(1-e^{-H/t_{i}}))\frac{\sin(\theta )}{\left(1-2\sin^{2}(\theta )\right)}\times \frac{l}{l_{e}w(h+d)}$$

where l is the distance from the load point of application to the support, l_e_ and w are the effective length and width of the CFRP bonding, respectively, h is the specimen height.

### 4.3. Maximum Strain Distribution in CFRP–Concrete Interface

**Figure 11:**
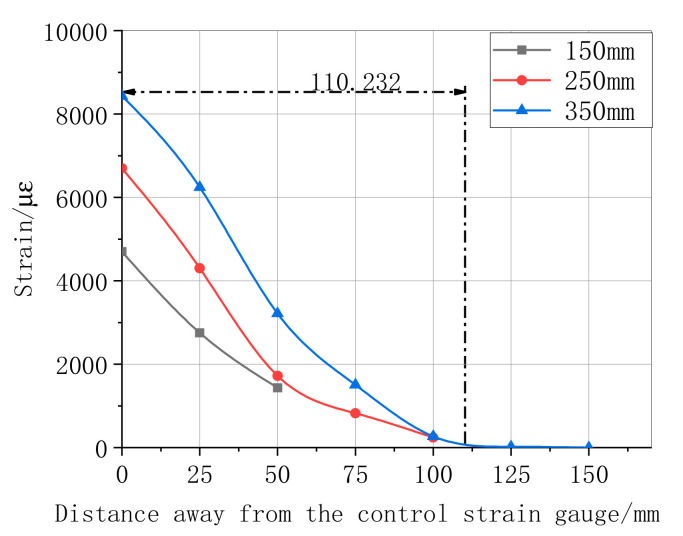
Ultimate strain of different lengths of CFRP bonding.

**Figure 12:**
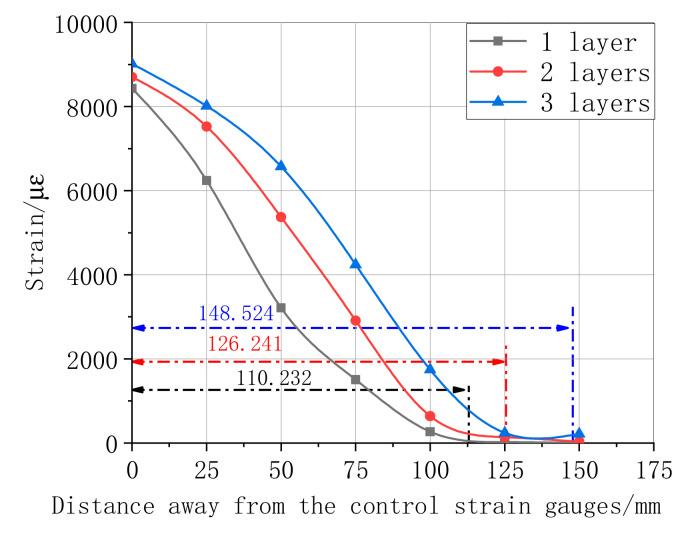
Ultimate strain of different CFRP layers of bonding length 350 mm.

**Figure 13:**
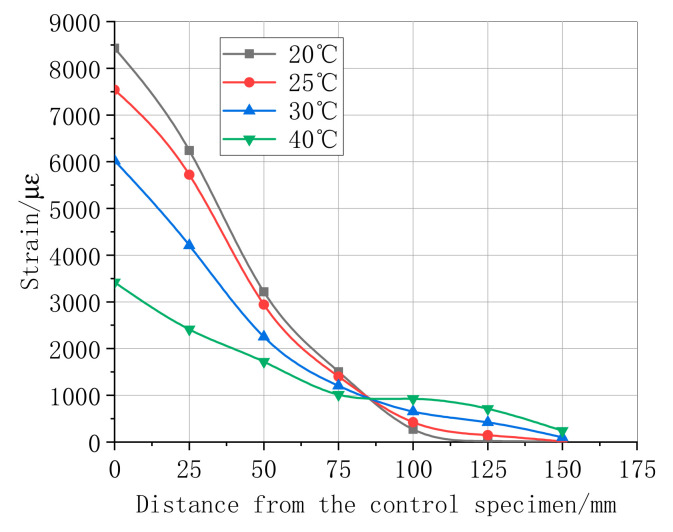
Ultimate strain of CFRP length 350 mm under different temperature.

**Figure 14:**
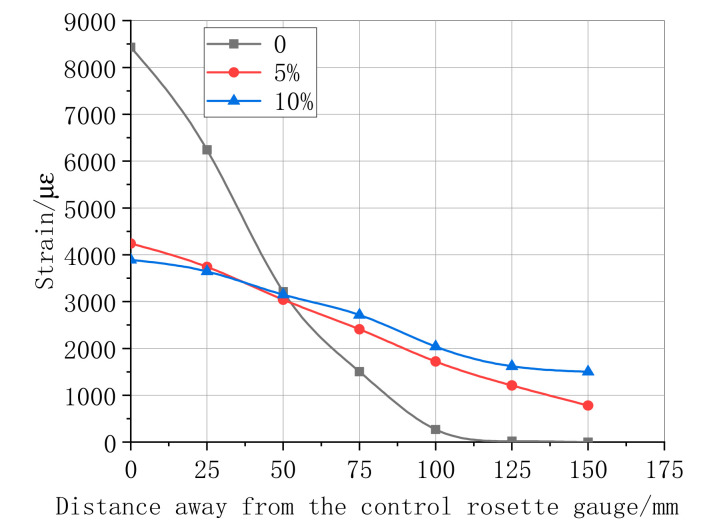
Ultimate strain of CFRP length 350 mm under different humidity.

The maximum shear stress distributions in the specimen interface of the bonded CFRP when the load achieved the limit value are shown in Figure 11, Figure 12, Figure 13 and Figure 14. Figure 11 summarizes the experimental data of the maximum shear stress for a differently bonded length of the CFRP adhesive. The maximum shear stress decreased with an increase in the distance from the first central strain gauge to the tested strain gauge; however, the rate decreased when the location of the bonded strain gauge changed from 50 mm to 120 mm. The experimental result showed that the strain appeared only near the free end of CFRP in the bonded length of 150 mm and 250 mm. These results prove that the effective bond length value was above 250 mm. According to the strain curve of length 350 mm, the effective bond length in this study was 110.232 mm. Following the relationship of the strain–distance curves of different layers, as shown in Figure 12, the first central maximum strain of the bonding layer 3 was higher than that of the bonding layers 1 and 2. Moreover, the range of the maximum strain, from a distance rate of 0 and 100, was approximately linear. During this process, one of the maxima tested strain was always a strain of the bonding layer 3. Next, the maximum strain of the layers decreased slowly, whereas the effective bond length increased substantially with the rate of CFRP layers, which indicates that the maximum strain is more sensitive to high layers.

Figure 12 and Figure 13 show the maximum shear stress of the specimens for different humidity and temperature factors. With an increase in temperature, the maximum stress, tested at the first central strain gauge, reduced significantly. Moreover, with the recent distance from the tested stress to the free end, the maximum shear stress gradually decreased until zero. The effective bond length of the CFRP–concrete under different humidity and temperature conditions are compared in Figure 12 and Figure 13. With an increase in the humidity and temperature, the numerical values of the effective bond length under temperature 20 °C and 40 °C and a humidity of 5% and 10% were higher than the bonding length because the strain values at the free end of the CFRP were not zero.

The maximum shear strain is evaluated by Equations (5) and (6) to accurately predict the effective bond length beyond the free end of the CFRP after logistic function fitting in different hygrothermal scenarios. Further, the parameters of the equation are calculated as summarized in Table 6 and Table 7.

(5) $$\varepsilon_{\max}{}^{\left(T\right)}=\frac{A_{3}-A_{4}}{1+{\left(\frac{l_{\mathrm{eT}}}{l_{0}}\right)}^{p}}+A_{4}$$

(6) $$\varepsilon_{\max}{}^{\left(H\right)}=a\;+\;b\times l_{\mathrm{eH}}$$

where $A_{3}$ and $A_{4}$ are the strain of the first and last strain gauge, respectively, and P is the index of the effective bond length and different thermal scenarios, $l_{\mathrm{eT}}$ and $l_{\mathrm{eH}}$ are the effective bond lengths under different hygrothermal scenarios, $l_{0}$ is the fixed length of effective bond length. a  is the strain of the first strain gauge, b is the linear slope of maximum strain and the effective bond length.

According to the parameter values of Table 6 and Table 7, and the conditions of the effective bonding length, when the strain value is 0, the effective bonding length can be calculated by following Table 6 and Table 7. With an increase in temperature, the effective bond length calculated by Equation (7) also increases. In Table 7, with an increase in humidity, the effective bond length calculated by Equation (8) increases. After the fitting model, the relationship between the effective bond length and temperature/humidity is obtained, respectively, as follows:

(7) $$l_{e}=657T^{3}-21T^{2}+0.23T^{3}-6378$$

(8) $$l_{e}=347-236e^{-H/15}$$

### 4.4. Relationship between Slip Distribution in CFRP–Concrete Interface

The displacement in the CFRP–concrete interface represents the relation slip, which led to failure in the model concrete lining strengthened by CFRP. The relationship between the slip in the middle of the two strain gauges can be described by Equation (9) [31].

(9) $$s_{i+\frac{1}{2}}=s_{0}+\sum_{m=1}^{i-1}\frac{1}{2}(\varepsilon_{m}+\varepsilon_{m+1})\Delta l-\frac{1}{4}(\varepsilon_{i}+\varepsilon_{i+1})\Delta l$$

where S_i+1/2_ is the slip of the adhesive and the concrete located in the middle of the i th and i + 1-th electric resistance strain gauges (i = 1 in the center of the specimen), $\varepsilon_{i}$ and Δl are the strain values of the i-th gauge and the space between two gauges, respectively, $s_{0}$ is the slip at the center of the specimen as follows:

(10) $$s_{0}=\frac{\tau_{\max}}{{\beta K}_{i}}$$

where β is the interface stiffness coefficient, and its value is 1.347. Let $K_{i}$ be the interface stiffness of the internal concrete–CFRP, and $K_{i}=\frac{K_{a}K_{c}}{K_{a}+K_{c}}$, where $K_{c}$ and $K_{a}$ are the interface stiffness of the concrete and adhesive, respectively. These parameters are calculated as follows:

(11) $$K_{c}=\frac{G_{c}}{t_{c}}$$

(12) $$K_{a}=\frac{G_{a}}{t_{a}}$$

where $G_{c}$ and $G_{a}$ are the shear modulus of the concrete and adhesive, and their values are 1.2 × 10^10^ (N/m^2^) and 1.07 × 10^13^ (N/m^2^), respectively. t_c_ and t_a_ are the failure thickness of the concrete and the adhesive, respectively. Moreover, t_c_ is 0.25 mm and t_a_ is 0.53 mm.

**Figure 15:**
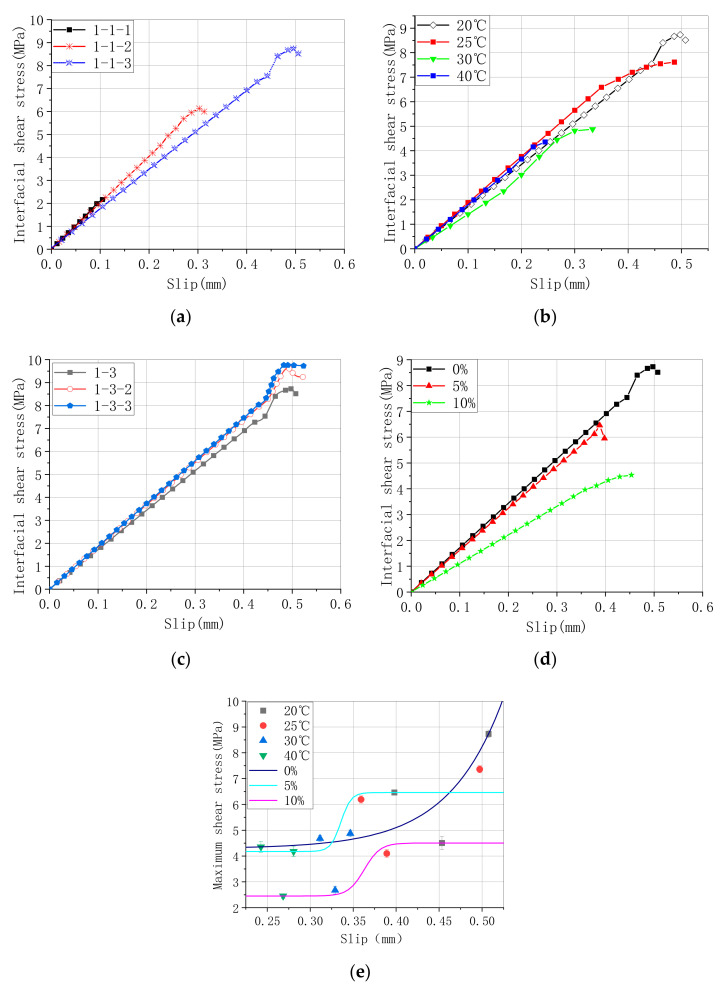
Interfacial shear stress and slip of CFRP bonding concrete arch. (**a**) Shear stress and slip curves for different CFRP lengths, (**b**) shear stress and slip curves for different, T.; (**c**) shear stress and slip curves for different CFRP layers, (**d**) shear stress and slip curves for different H, (**e**) maximum interfacial shear stress and slip curves for hygrothermal coupling.

When considering Equation (9) and the shear stress (Equations (3) and (4)), the relationship between the interfacial shear stress and the slip of the concrete arch strengthened by CFRP is shown in Figure 15 and Table 8. According to the slip limit in Table 8, the slip relation under the standard conditions and different circumstances can be obtained as follows:

(13) $$\begin{array}{l} \frac{s}{s_{0}}=0.48+\sum_{i=1}^{2}-0.11(1-e^{\frac{-l}{-127}})\;\\ \frac{s}{s_{0}}=\frac{1-1.03}{1+e^{10}{}^{\left(n-1.55\right)}}+1.03\\ \frac{s}{s_{0}}=\frac{1-0.48}{1+e^{}{}^{\left(T-29.1\right)}}+0.48\\ \frac{s}{s_{0}}=\frac{1-0.11}{1+e^{}{}^{{\left(H-2.85\right)}^{3}}}+0.11 \end{array}$$

where l is the bonding length of CFRP, n is the bonding layers.

Figure 15 shows the relationship between shear stress and the slip under different environments. For the strengthened specimens of differently longitudinal CFRP, the shear stress is linearly dependent on the slope as the load increases until it fails. Furthermore, the relationship between the ultimate slope and the length is almost proportional. With an increase in the number of CFRP layers, the stress–slope relation is gradually nonlinear after the load value reaches 80%, and the stress–slope curve is more nonlinear with increasing temperature or humidity than the curve shape with an increase in number and length of layers [32,33]. However, increasing the temperature is more effective than increasing the humidity because of the nonlinearity of the stress–slope curve.

## 5. Ultimate Bearing Capacity of Strengthening Shield Segments on the Shear-Slip of CFRP-Reinforced Concrete Arch Sections

### 5.1. Derivation of Ultimate Bearing Capacity Formulation

**Figure 16:**
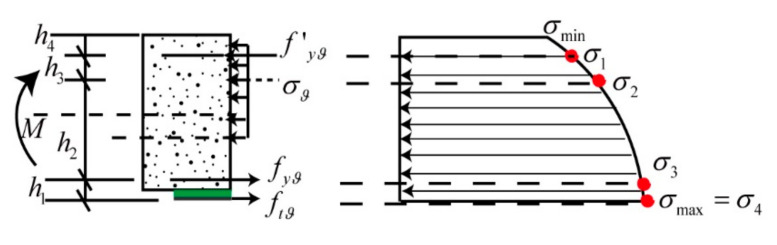
Tangential stress analysis.

To estimate the ultimate bearing capacity [34,35,36,37], maximum deflection, and flexural strength of damaged shield tunnel segment strengthening with CFRP, the tangential stress of the strengthening shield segments was analyzed as shown in Figure 16 [38,39,40]. The maximum and minimum values of the tangential stress can be calculated using the equations in Appendix B [41,42].

(14) $$\sigma_{\max}=-\frac{r_{5}{}^{2}+r_{1}{}^{2}}{r_{5}{}^{2}-r_{1}{}^{2}}P_{};\;\;\;\sigma_{\min}=-\frac{2r_{1}{}^{2}}{r_{5}{}^{2}-r_{1}{}^{2}}P_{}$$

The analysis assumes that the reinforced concrete and the CFRP work together in Figure 16. The different materials’ strengthened shield forces can be calculated following the equation of strain compatibility given by [43,44,45,46,47]:

(15) $$\begin{array}{l} f_{t}{}_{\vartheta }=\frac{-2P_{12}r_{2}{}^{2}}{r_{2}{}^{2}-r_{1}{}^{2}}>\sigma_{\max}=\sigma_{4}\\ f_{y}{}_{\vartheta }=\frac{r_{2}{}^{2}+r_{3}{}^{2}}{r_{3}{}^{2}-r_{2}{}^{2}}P_{12}-\frac{r_{3}{}^{2}}{r_{3}{}^{2}-r_{2}{}^{2}}P_{32}>\sigma_{3}\\ \sigma_{\vartheta }=\frac{r_{3}{}^{2}+r_{4}{}^{2}}{r_{4}{}^{2}-r_{3}{}^{2}}P_{32}-\frac{r_{4}{}^{2}}{r_{4}{}^{2}-r_{2}{}^{2}}P_{43}>\sigma_{2}\\ {f’}_{y}{}_{\vartheta }=\frac{r_{5}{}^{2}+r_{4}{}^{2}}{r_{5}{}^{2}-r_{3}{}^{2}}P_{43}-\frac{-2r_{5}{}^{2}}{r_{5}{}^{2}-r_{3}{}^{2}}P_{}>\sigma_{1} \end{array}$$

where $r_{1},r_{2},r_{3},r_{4}\;\mathrm{and}\;r_{5}$ are the radiuses from the CFRP, tensile steel, concrete, and compressional steel to the circle center of the shield tunnel, respectively; their relationships are presented by

(16) $$r_{5}=h_{4}+r_{4}=(h_{4}+h_{3})+r_{3}=(h_{4}+h_{3}+h_{2})+r_{2}=(h_{4}+h_{3}+h_{2}+h_{1})+r_{1}$$

$P,\;P_{43},\;P_{32}\;\mathrm{and}\;P_{12}$ correspond to the surface pressure forces acting simultaneously, which are calculated as

(17) $$P_{12}=\frac{u_{1c}}{u_{t}+u_{1c}}P_{32};\;\;\;P_{32}=\frac{u_{y}}{u_{1c}+u_{y}-u_{1c}u_{1c}/(u_{t}+u_{1c})}P_{43};\;\;\;P_{43}=\frac{u_{y’}}{u_{2c}+u_{y’}-u_{1c}u_{y}/(u_{1c}+u_{y})}P_{}$$

where $u_{t}$ is the radial displacement of the adhesive and CFRP, $u_{1c}$ is the radial displacement of the concrete surface from the bonding CFRP to the tensile steel, and $u_{y}$ and $u_{y’}$ are the radial displacements of the compression and tensile steel, respectively. They are calculated according to China national standard (GB50010-2010) and (GB50384-2016) as:

(18) $$\begin{array}{l} u_{y}=(1+\mu_{g})(\frac{1+h_{1}{}^{2}-2\mu_{g}h_{1}{}^{2}}{h_{1}{}^{2}-1})\frac{r_{1}}{E_{f}}\\ u_{f’}=(1+\mu_{g})(\frac{1+{\left(h_{3}+h_{4}\right)}^{2}-2\mu_{g}{\left(h_{3}+h_{4}\right)}^{2}}{{\left(h_{3}+h_{4}\right)}^{2}-1})\frac{r_{5}}{E_{f’}}\\ u_{1c}=(1+\mu_{c})(\frac{1+h_{1}{}^{2}-2\mu_{c}h_{1}{}^{2}}{h_{1}{}^{2}-1})\frac{r_{1}}{E_{c}}\\ u_{2c}=(1+\mu_{c})(\frac{1+{\left(h_{3}+h_{4}\right)}^{2}-2\mu_{c}{\left(h_{3}+h_{4}\right)}^{2}}{{\left(h_{3}+h_{4}\right)}^{2}-1})\frac{r_{5}}{E_{c}} \end{array}$$

where μ is the Poisson ratio, and subscripts g and c stand for steel and concrete, respectively, E is the Young’s modulus, h_i_ is the distance of the different structure, and i ranges from 1 to 5. In different tunnel environments, the effect of CFRP bonding has a great influence. Finally, $u_{t}$ is the result of Equation (10).

For a CFRP-strengthened segment joint as shown in Appendix B, Figure 1b, the segment joint is forced to be connected using two bolts; therefore, the concrete compressive stress $\sigma_{\vartheta }$ of the segment joint is zero. It is naturally satisfied that the segment resistance always achieves the design requirements because the internal forces of bolts resist the internal forces of the segment joints.

### 5.2. Comparison of Analysis and Test Results

Table 9 summarizes the CFRP debonding values for different structural sites of the shield segment, when H = 0%, 5%, and 10%. The values include predicted load (P_1_), ultimate load (P_2_), and the mechanical parameters of reinforced concrete, which are the tensile stress of CFRP ($f_{t}{}_{\vartheta }$), the tensile stress of steel ($f_{y}{}_{\vartheta }$), the compressive stress of concrete ($\sigma_{\vartheta }$), and compressive stress of steel (${f’}_{y}{}_{\vartheta }$).

**Figure 17:**
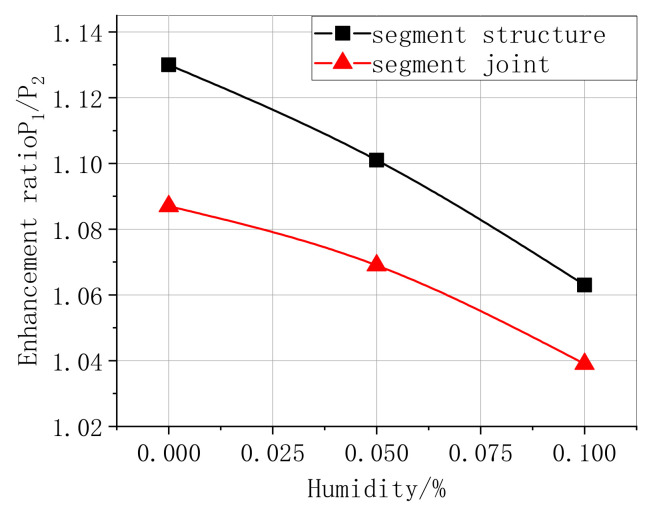
Ultimate load enhancement ratio of the CFRP bonding segment structure and joint.

**Figure 18:**
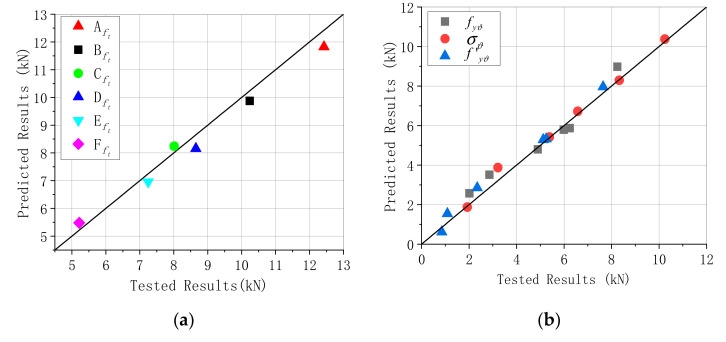
Comparison of the analytical and tested results. (**a**) Prestrengthening CFRP, (**b**) poststrengthening reinforced concrete.

The bearing capacity enhancement ratio of CFRP bonding segment structure and joint, not subjected to humidity, were 1.13 and 1.087, respectively. Moreover, Table 9 lists that the effect of the CFRP-strengthened segment structure was evident. The enhancement ratio reduced apparently by 1.13, 1.101, and 1.063 (segment structure), and it reduced sharply by 1.087, 1.069, and 1.039 (segment joint) when increasing the humidity from 0% to 10%; see Table 9 and Figure 17. Based on the shear-slip experiment of CFRP-reinforced arch sections and considering the effect of humidity on the debonding strength, we calculated the values of the CFRP, steel, and concrete strain in Table 9. Figure 18 and Table 10 presents a comparison between the computational values and internal forces of CFRP, steel, and concrete obtained in the experimental measurements. The predicted results were similar to the tested internal force, whereas the calculated results were higher than the measured force when the humidity was 0% and 5%; nevertheless, the calculated results were smaller when the humidity was 10%.

## 6. Conclusions

This study examined the ultimate bearing capability of a CFRP-strengthened prepressing damage shield segment with respect to the shear-slip of CFRP bonding. From the study, the following conclusions can be drawn:

The test results of the shear-slip of CFRP bonding showed that the parameters of CFRP, which included length, layers, humidity, and temperature, had great influence on the binding effect. They specifically included the following points:

1. The limit load as the length and layers increased showed a substantial effect on the flexural and sheared behavior of concrete arch sections reinforced with CFRP strengthening. However, the enhancement ratio of the limit load was more effective than the layers because of the increment in the CFRP length.
2. The increment in the temperature caused a substantial reduction in the load limit of the strengthened specimens, and the limit load was similar to that of the specimens with an increase in humidity. The combined sustained humidity and temperature as a damaged factor considerably reduced the peak strength of the beams with CFRP strengthening.
3. With regard to the maximum shear stress and strain, the slip relation under different factors was obtained. Adding layers and increasing the length caused the relation curve to create shear stress and increase the slope linearly; the load-slip curve changed from linear to nonlinear in the hygrothermal environment.

According to the theory of elasticity for composite cylinder structures, a general solution of the stress function was developed as a function of the curvature radius along with the characteristics of the compatibility equation. Moreover, the stress function of the cross-section was obtained; thus, the stress of CFRP, steel, and concrete was calculated using the equation of composite structures.

The predicted results, based on the stress formula of elasticity, were similar to the internal force compared to the experimental results. The calculated results were higher than the measured force when the humidity was 0% and 5%; however, the calculated results were smaller when the humidity was 10%.

## Figures and Tables

**Table 1:** Mechanical parameters of the CFRP and adhesive.

CFRP	Elastic Modulus E_f_(N/mm^2^)	$\mathrm{Maximum}\;\mathrm{Strain}\;\varepsilon_{\mathrm{fu}}$		Ultimate Strength $f_{\mathrm{fu}}\left(\mathrm{MPa}\right)$	
JGN	2.5 × 10^5^	1.5%		3700	
**Adhesive**	**$\mathrm{Elastic}\;\mathrm{Modulus}\;E_{e}\cdot \mathrm{GPa}\cdot$**	**Ultimate Strength** $f_{e}\left(\mathrm{MPa}\right)$	**Elongation %**		**Bending Strength** $f_{e}{}_{f}\left(\mathrm{MPa}\right)$
JGN-C	26700	52	1.7		76

**Table 2:** Slip testing parameters of CFRP-reinforced arch concrete sections.

Model Number	CFRP		Environmental Parameter		Model Number	CFRP		Environmental Parameter	
	Length	Layers	T/°C	H/%		Length	Layers	T/°C	H/%
1-1	150	1	20	0%	2-1	350	1	20	5%
1-2	250	1	20	0%	2-2	350	1	25	5%
1-3	350	1	20	0%	2-3	350	1	35	5%
1-3-2	350	2	20	0%	2-4	350	1	40	5%
1-3-3	350	3	20	0%	3-1	350	1	20	10%
1-4-2	350	1	25	0%	3-2	350	1	25	10%
1-4-3	350	1	35	0%	3-3	350	1	35	10%
1-4-4	350	1	40	0%	3-4	350	1	40	10%

**Table 3:** Limit load of damaged specimens.

Model Number	CFRP		Environmental Parameter		Loading/kN	Model Number	CFRP		Environmental Parameter		Loading/kN
	Length	Layers	T/°C	H/%			Length	Layers	T/°C	H/%	
1-1	150	1	20	0%	15.0	2-1	350	1	20	5%	26.2
1-2	250	1	20	0%	17.1	2-2	350	1	25	5%	25.7
1-3	350	1	20	0%	30.1	2-3	350	1	35	5%	20.6
1-3-2	350	2	20	0%	34.2	2-4	350	1	40	5%	20.0
1-3-3	350	3	20	0%	40.2	3-1	350	1	20	10%	19.2
1-4-2	350	1	25	0%	28.7	3-2	350	1	20	10%	17.3
1-4-3	350	1	35	0%	20.9	3-3	350	1	35	10%	12.1
1-4-4	350	1	40	0%	19.9	3-4	350	1	40	10%	11.6

**Table 4:** Related coefficients for limit load and temperature.

Humidity/%	T_0_	A_1_	A_2_	R^2^/%
0	27.4	30.0	19.9	98.6
5	27.7	26.2	20.1	97.8
10	27.2	19.2	11.6	93.5

**Table 5:** Related coefficients for limit load and humidity.

Temperature/T °C	L_0_	i	A_i_	t_i_	R^2^/%
20	30.0	2	2.03	−8.29	99.7
25	28.7	2	0.56	−4.19	96.7
30	20.9	2	0.01	−1.49	97.8
40	19.9	2	1.16 × 10^−6^	−0.78	92.8

**Table 6:** Parameters for the relationship between the maximum shear stress and temperature.

T °C	A_3_	A_4_	p	l_0_	R^2^*/%*	l_eT_/mm
20	8431	0	3	41.8	99.8	110
25	7542	12	3	42.5	96.2	363
30	6024	10	3	40.2	97.5	409
40	3421	5	3	50.2	98.5	563

**Table 7:** Parameters for the relationship between the maximum shear stress and humidity.

Humidity	a	b	R^2^*/%*	l_eH_/mm
0%	7169	−58.1	87.7	110
5%	4245	−23.9	99.5	177
10%	3972	−17.6	98.1	226

**Table 8:** Maximum interfacial shear stress and slip.

Model Number	CFRP		T/°C	H/%	d/mm	$\theta /10^{-1}\pi$	S_0_ 10^−1^ mm	$l_{e}\left(\mathrm{mm}\right)$	$\tau_{1\max}/\mathrm{MPa}$	S 10^−1^ mm
	Length/mm	Layers								
1-1	150	1	20	0	12.0	0.94	0.34	50	2.16	1.05
1-2	250	1	20	0	20.0	1.33	0.96	75	6.13	3.03
1-3	350	1	20	0	30.8	1.50	1.36	110	8.73	5.07
1-3-2	350	2	20	0	25.1	1.37	1.49	126	9.61	5.22
1-3-3	350	3	20	0	14.9	1.01	1.52	148	9.76	5.23
1-4-2	350	1	25	0	31.2	1.54	1.15	150	7.36	4.97
1-4-3	350	1	30	0	35.6	1.59	0.76	150	4.88	3.46
1-4-4	350	1	40	0	40.3	1.67	0.68	150	4.36	2.42
2-1	350	1	20	5	33.2	1.57	1.01	150	6.46	3.97
2-2	350	1	25	5	34.2	1.58	0.96	150	6.19	3.59
2-3	350	1	30	5	37.9	1.64	0.73	150	4.68	3.11
2-4	350	1	40	5	42.6	1.69	0.65	150	4.18	2.81
3-1	350	1	20	10	35.3	1.58	0.70	150	4.50	4.53
3-2	350	1	25	10	36.0	1.63	0.64	150	4.09	3.89
3-3	350	1	30	10	39.2	1.66	0.42	150	2.68	3.29
3-4	350	1	40	10	45.3	1.81	0.38	150	2.45	2.69

**Table 9:** Analytical and experimental results of the segment structure and joint under different humidity values.

Spe.	Pre-Load	Ultimate Load	Ratio	Analytical Results on Slope of CFRP/MPa				Test Results of Segment Strengthened by CFRP/MPa			
	P_1_	P_2_	P_2_/P_1_	$f_{t}{}_{\vartheta }$	$f_{y}{}_{\vartheta }$	$\sigma_{\vartheta }$	${f’}_{y}{}_{\vartheta }$	$f_{t}{}_{\vartheta 2}$	$f_{y}{}_{\vartheta 2}$	$\sigma_{\vartheta 2}$	${f’}_{y}{}_{\vartheta 2}$
A	301	340	1.13	12.4	8.24	10.2	7.6	11.8	8.9	10.4	7.9
B	301	327	1.09	8.7	6.24	6.6	5.2	8.2	5.9	6.7	5.3
C	289	318	1.10	10.2	5.98	8.3	5.1	9.9	5.8	8.3	5.3
D	289	309	1.07	7.2	4.89	5.3	2.3	6.9	4.8	5.4	2.9
E	273	290	1.06	8.0	2.86	3.2	1.1	8.2	3.5	3.8	1.5
F	273	283	1.04	5.2	2.01	1.9	0.9	5.5	2.6	1.8	0.6

**Table 10:** Comparison between the analytical and experimental results.

Spe.	$f_{t}{}_{\vartheta }/f_{t}{}_{\vartheta 2}$	$f_{y}{}_{\vartheta }/f_{y}{}_{\vartheta 2}$	$\sigma_{\vartheta }/\sigma_{\vartheta 2}$	${f’}_{y}{}_{\vartheta }/{f’}_{y}{}_{\vartheta 2}$
A	1.05	0.92	0.99	0.96
B	1.06	1.06	0.98	0.98
C	1.04	1.03	1.00	0.97
D	1.04	1.02	0.99	0.82
E	0.97	0.81	0.83	0.70
F	0.95	0.78	1.03	1.39

**Figure A1:**
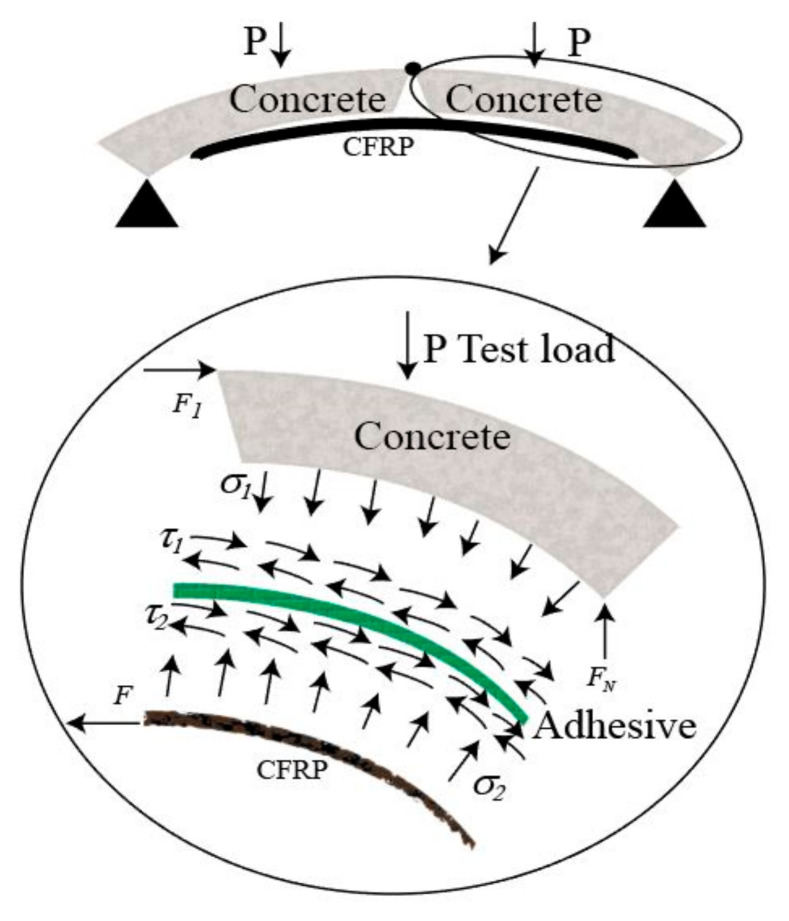
Relation between load and shear.

**Figure A2:**
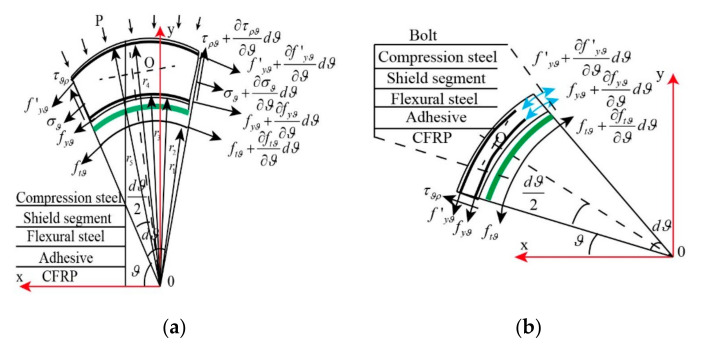
Force analysis diagram of the shield segment strengthened by CFRP. (**a**) Segment structure strengthened by CFRP, (**b**) segment joint strengthened by CFRP.

## Appendix A. The Maximum Shear Stress of CFRP-Reinforced Concrete Arch Sections

In the test, the hydraulic press load is an active force; therefore, the limit load is related to the maximum shear stress. When the load was increased, the CFRP debonding always initiated in the middle of the two specimens near the gap area of the adhesive and concrete, where the tensile behavior of CFRP was observed. Moreover, as the load increased further, the two strengthened specimens rotated by the supporting point where the two specimens approached each other, and the CFRP debonding further developed because of failure, as can be seen in Figure A1.

According to the equilibrium limit condition form analytic free-body methods, shown in Figure A1, the right side of the entire strengthened specimen is studied separately. Accordingly, the tension of the CFRP can be calculated using:

(A1) $$F=\frac{l}{h+d}P$$

where F and P are the CFRP tensile strength and test load of the mechanical and hydraulic press, respectively, l is the distance from the load point of application to the support, and h is the specimen height. The shear and normal stress equations of the CFRP internal area and the adhesive are given as:

(A2) $$\begin{array}{l} \sigma_{2}\sin(\theta )=\tau_{2}\cos(\theta )\;\;\;\\ F+\tau_{2}l_{e}w\sin(\theta )=\sigma_{2}l_{e}w\cos(\theta )\; \end{array}$$

where l_e_ and w are the effective length and width of the CFRP bonding, respectively, $\sigma_{2}$ and $\tau_{2}$ are the stress–strain and maximum shear, respectively, and θ is the slope of the two strengthened specimens during the loading process. Equation (A3) is solved by combining them; thus, the CFRP shear stress and adhesive can be written as

(A3) $$\tau_{2}=\frac{F\sin(\theta )}{l_{e}w[1-2\sin^{2}(\theta )]}$$

In this study and several other studies [SiO_2_ adhesive], the adhesive showed ductile shear deformation caused by the apparent shear stress. The maximum shear stress at the interface of the adhesive is related to the adhesive tensile strength, with a correction coefficient of 0.5 [30]. Consequently, the maximum shear stress $\tau_{1}$ of the concrete and adhesive can be calculated as:

(A4) $$\tau_{1}=0.5\tau_{2}$$

and the relationship of the maximum shear stress $\tau_{1}$ and the load limit is calculated using:

(A5) $$\tau_{1}=\frac{0.5P\sin(\theta )}{(1-2\sin^{2}(\theta ))\cdot h+d\cdot w}•\frac{l}{l_{e}}$$

## Appendix B. The Maximum and Minimum Values of CFRP-Strengthened Tunnel Segmental Lining

Figure A2a presents the simplified force diagram versus the infinitesimal element of arch dϑ and the radius vector ρ of a point in polar coordinates of a shield structural segment strengthened by CFRP. All force projects to the tangential axis at the differential center of point O. The sum of all forces in the tangential axis direction can be expressed as

(A6) $$\begin{array}{l} \frac{\partial f_{t}{}_{\vartheta }}{\partial \vartheta }d\vartheta d\rho \cos\frac{d\vartheta }{2}+\;\frac{\partial f_{y}{}_{\vartheta }}{\partial \vartheta }d\vartheta d\rho \cos\frac{d\vartheta }{2}+\frac{\partial \sigma_{\vartheta }}{\partial \vartheta }d\vartheta d\rho \cos\frac{d\vartheta }{2}+\frac{\partial {f’}_{y}{}_{\vartheta }}{\partial \vartheta }d\vartheta d\rho \cos\frac{d\vartheta }{2}\;+\\ (\tau_{\vartheta \rho }+\frac{\partial \tau_{\vartheta \rho }}{\partial \vartheta }d\vartheta )d\rho \sin(\frac{d\vartheta }{2}){+\tau }_{\vartheta \rho }d\rho \sin(\frac{d\vartheta }{2})=0 \end{array}$$

Moreover, the sum of all forces in the radial axis is presented as in

(A7) $$\begin{array}{l} -(f_{t}{}_{\vartheta }+\frac{\partial f_{t}{}_{\vartheta }}{\partial \vartheta }d\vartheta )d\rho \sin\frac{d\vartheta }{2}-f_{t}{}_{\vartheta }d\rho \sin\frac{d\vartheta }{2}-(f_{y}{}_{\vartheta }+\frac{\partial f_{y}{}_{\vartheta }}{\partial \vartheta }d\vartheta )d\rho \sin\frac{d\vartheta }{2}-\\ f_{y}{}_{\vartheta }d\rho \sin\frac{d\vartheta }{2}-(\sigma_{\vartheta }+\frac{\partial \sigma_{\vartheta }}{\partial \vartheta }d\vartheta )d\rho \sin\frac{d\vartheta }{2}-\sigma_{\vartheta }d\rho \sin\frac{d\vartheta }{2}-({f’}_{y}{}_{\vartheta }+\frac{\partial {f’}_{y}{}_{\vartheta }}{\partial \vartheta }d\vartheta )\\ d\rho \sin\frac{d\vartheta }{2}-{f’}_{y}{}_{\vartheta }d\rho \sin\frac{d\vartheta }{2}+(\tau_{\vartheta \rho }+\frac{\partial \tau_{\vartheta \rho }}{\partial \vartheta }d\vartheta )d\rho \cos(\frac{d\vartheta }{2})-\tau_{\vartheta \rho }d\rho \cos(\frac{d\vartheta }{2})=0 \end{array}$$

As the arch element dϑ is very small, we can approximate $\sin\frac{d\vartheta }{2}$ to $\frac{d\vartheta }{2}$, and $\cos(\frac{\vartheta }{2})$ to 1. The differential equation of the shield structural segment strengthened by CFRP can be expressed as:

(A8) $$\frac{\partial f_{t}{}_{\vartheta }}{\partial \vartheta }+\frac{\partial f_{y}{}_{\vartheta }}{\partial \vartheta }+\frac{\partial \sigma_{\vartheta }}{\partial \vartheta }+\frac{\partial {f’}_{y}{}_{\vartheta }}{\partial \vartheta }+\tau_{\vartheta \rho }+\frac{\partial \tau_{\vartheta \rho }}{2\partial \vartheta }d\vartheta =0$$

(A9) $$\frac{\partial \tau_{\vartheta \rho }}{\partial \vartheta }-(f_{t}{}_{\vartheta }+f_{y}{}_{\vartheta }+\sigma_{\vartheta }+{f’}_{y}{}_{\vartheta })-\frac{1}{2}(\frac{\partial f_{t}{}_{\vartheta }}{\partial \vartheta }+\frac{\partial f_{y}{}_{\vartheta }}{\partial \vartheta }+\frac{\partial \sigma_{\vartheta }}{\partial \vartheta }+\frac{\partial {f’}_{y}{}_{\vartheta }}{\partial \vartheta })=0$$

where $f_{t}{}_{\vartheta },f_{y}{}_{\vartheta },\sigma_{\vartheta },{f’}_{y}{}_{\vartheta }\;\mathrm{and}\;\tau_{\vartheta \rho }$ are the CFRP tangential stress, tensile steel, concrete and compressional steel, and structural shear stress, respectively. The normal and shear stress of the arch-bending element can be expressed as a function φ(ρ, ϑ) as follows:

(A10) $$f_{t}{}_{\vartheta }+f_{y}{}_{\vartheta }+\sigma_{\vartheta }+{f’}_{y}{}_{\vartheta }=\frac{\partial^{2}\varphi }{\partial \rho^{2}}$$

(A11) $$\tau_{\vartheta \rho }=-\frac{\partial }{\partial \rho }(\frac{1}{\rho }\frac{\partial \varphi }{\partial \vartheta })$$

They require that the stress function φ(ρ, ϑ) should be satisfied by compatible equations as can be seen in:

(A12) $${\left(\frac{\partial^{2}}{\partial \rho^{2}}+\frac{1}{\rho }\frac{\partial }{\partial \rho }+\frac{1}{\rho^{2}}\frac{\partial^{2}}{\partial \vartheta^{2}}\right)}^{2}\varphi =0$$

As the shield lining is asymmetric, the error range is 0.15 (radians), which is negligible, and the compatible equations are simplified to:

(A13) $$\frac{\partial \varphi }{\partial \vartheta }=0,\;\;\;\;\;\;\;\;\frac{1}{\rho }\frac{d^{2}}{d\rho }\left\{\rho \frac{d}{d\rho }[\frac{1}{\rho }\frac{d}{d\rho }(\rho \frac{d\varphi }{d\rho })]\right\}=0$$

Moreover, the stress function φ(ρ, ϑ) can be represented as:

(A14) $$\varphi (\rho )=A\ln\rho +{B\rho }^{2}\ln\rho +{C\rho }^{2}+D$$

The shield lining strengthened by CFRP is subjected to a uniform pressure q*_2_* outside of the shield tunnel. Assuming that the outer radius of the shield lining is R, r is equal to the outer radius, and the boundary conditions for the stress field should satisfy:

(A15) $$\begin{array}{l} {\left(\tau_{\rho \vartheta }\right)}_{\rho =r_{1}}=\;0\;\;\;\;{\left(\tau_{\rho \vartheta }\right)}_{\rho =r_{1}}=\;0\;\\ {\left(\sigma_{\rho }\right)}_{\rho =r_{5}}=0\;\;\;\;\;{\left(\sigma_{\rho }\right)}_{\rho =r_{5}}=-P \end{array}$$

The parameters of the stress function φ(ρ, 0) are obtained only when the condition given by the boundary conditions is satisfied. That is:

(A16) $$B=0,\;\;\;A=\frac{r_{1}{}^{2}r_{5}{}^{2}q}{r_{5}{}^{2}-r_{1}{}^{2}},\;C=\frac{1}{2}\frac{-{\mathrm{qr}}_{5}{}^{2}}{r_{5}{}^{2}-r_{1}{}^{2}}$$

The maximum and minimum values of the tangential stress can be calculated using:

(A17) $$\sigma_{\max}=-\frac{r_{5}{}^{2}+r_{1}{}^{2}}{r_{5}{}^{2}-r_{1}{}^{2}}P_{};\;\;\;\sigma_{\min}=-\frac{2r_{1}{}^{2}}{r_{5}{}^{2}-r_{1}{}^{2}}P_{}$$
